# Giant superficial angiomyxoma of the male perineum: A case report

**DOI:** 10.3389/fsurg.2022.1010050

**Published:** 2023-01-06

**Authors:** Sheng Yan, Yuhua Zou, Xinzhi Liao, Cunzhi Zhong, Shengyin Liu, Sigen Huang, Junrong Zou, Quanliang Liu

**Affiliations:** ^1^ Department of Urology, The First Affiliated Hospital of Gannan Medical University, Ganzhou, China; ^2^ Department of Anesthesiology, Operation Room, The First Affiliated Hospital of Gannan Medical University, Ganzhou, China; ^3^ Institute of Urology, Gannan Medical University, Ganzhou, China

**Keywords:** superficial angiomyxoma, perineal tumor, case report, surgical removal, clinical pathology

## Abstract

Superficial angiomyxoma (SA) is a rare benign tumor that occurs either in the superficial dermis or subcutaneously. It often occurs in the trunk, neck, or limbs, and grows slowly. The diameter of the tumor is usually less than 5 cm. A giant SA of the perineum in men is very rare. We detailed the diagnosis and treatment of male patients with perineal SA and performed a literature review. We report a case of a 42-year-old male patient. He was admitted to hospital with a perineal mass found more than 1 year previously. A pelvic contrast-enhanced computed tomography scan in our hospital suggests that a round slightly hypointense foci of about 6.0 cm × 8.6 cm × 4.5 cm in size with still clear borders was seen below the penile corpus cavernosum in the perineum. We performed a perineal mass excision under continuous epidural anesthesia. A postoperative pathology report diagnosed perineal SA. There was no recurrence at follow-up for 27 months up to May 2022. Perineal SA is rare and should be combined with patient history and imaging to ensure complete excision of the mass margins. Adherence to long-term postoperative follow-up is the key to curing this case.

## Introduction

Superficial angiomyxoma (SA), also known as a cutaneous mucinous tumor, is a rare benign soft tissue tumor of the skin. SA was first reported by Carney et al. in 1985, with an incidence in the range of 0.008%–3% (1). Most SAs are isolated and can manifest themselves in association with Carney syndrome (2), which includes mucinous tumors, patchy skin pigmentation, and endocrine hyperfunctional disorders. Preoperative imaging examination has a certain significance for the estimation of tumor range and prognosis. However, imaging lacks typical features and a diagnosis is often made definitively by pathology. A giant SA in the perineum is extremely rare, and we report this case with the aim of providing a reference experience for clinical management.

## Case presentation

A male patient aged 42 years was admitted to the hospital after “finding a perineal mass for more than 1 year.” The patient complained that he found a mass the size of peanut rice in the perineum 1 year previously, without tenderness and discomfort from ulceration. At that time, he did not pay attention to it, and the mass then gradually increased. A color Doppler ultrasound in the local hospital showed a slightly hypoechoic mass in the perineum, which was not treated at that time. During the physical examination, a hard and fixed mass of about 8.5 cm × 6 cm was palpable in the patient's perineum.

**Figure 1:**
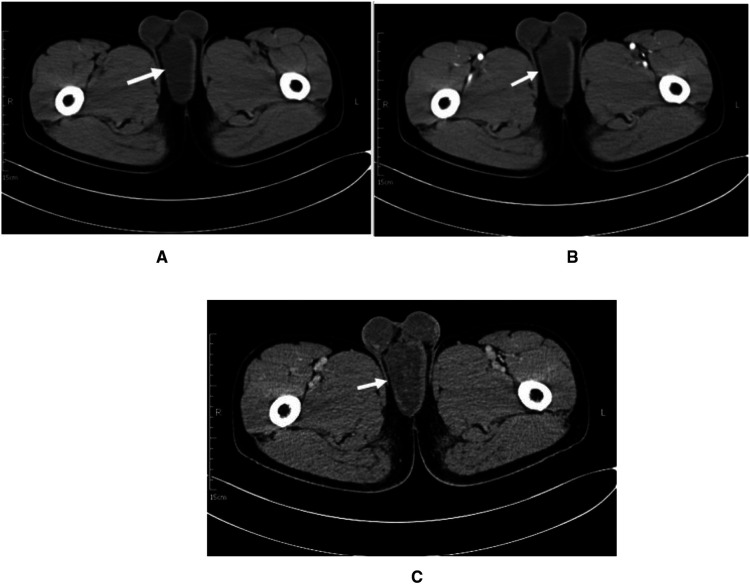
Patient imaging data. (**A**) CT plain axial image.(**B**) Arterial phase of the contrast. (**C**) Venous phase of the contrast.

To understand the nature, blood supply, and anatomical location of the mass, a contrast-enhanced computed tomography (CE-CT) scan of the pelvis was performed. A round slightly hypointense foci of about 6.0 cm × 8.6 cm × 4.5 cm in size with still clear borders was seen below the penile corpus cavernosum in the perineum (Figure 1A), with a CT value of approximately 17 HU. During the arterial phase of enhanced scanning, an arterial vessel was seen (Figure 1B). The enhancement of the lesion was not obvious, and uneven enhancement was seen in the venous phase (Figure 1C), with a CT value of approximately 31 HU. The prostate was small, the edge was smooth, and the density in the parenchyma was uniform. The shape, size, and density of the seminal vesicle gland were normal. The bladder was well filled. The bladder wall was smooth, without thickening or nodule protrusion. The bladder seminal vesicle angle was normal. There was no effusion or enlarged lymph nodes in the pelvic cavity. Initially, a large benign tumor in the perineum was considered, but the type of tumor was not yet clear. In the past, the patient had been in good health. No abnormality was found in his chest x-ray, ECG, or routine biochemical examination after admission.

**Figure 2:**
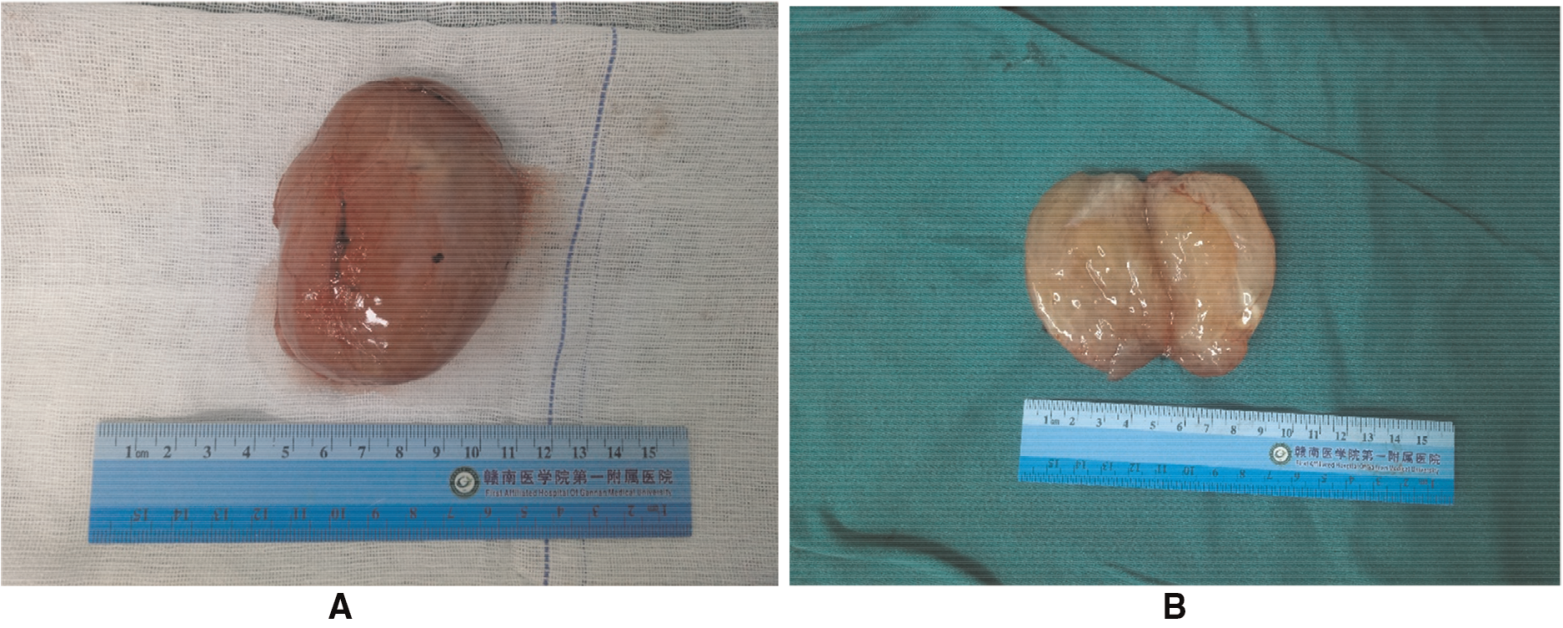
Patient tumor specimen. (**A**) The excised specimen was approximately 8.5 cm × 6 cm × 5 cm with clear margins. (**B**) The surface is grayish white, jelly-like, rubber-like.

**Figure 3:**
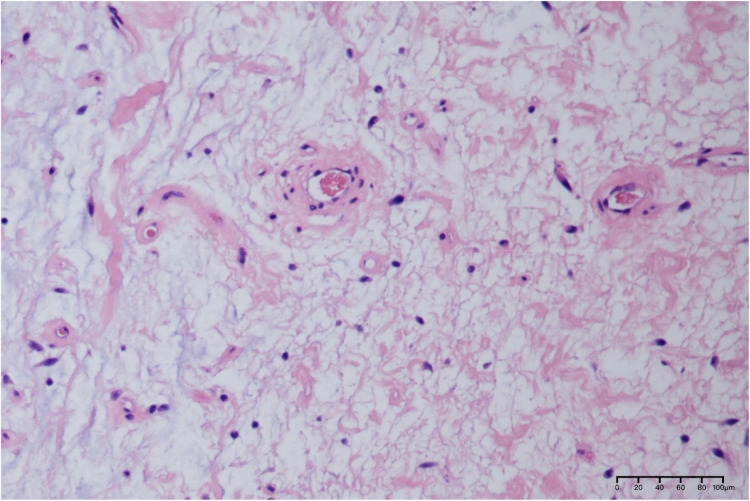
Pathological histological section of this case (HE × 200). Scattered spindle cells, stellate cells and perivascular inflammatory cells are visible against the background of a large amount of mucus stroma microscopically.

After communicating with the patient, he underwent a perineal mass resection under continuous epidural anesthesia. A lithotomy position and indwelling catheterization were used. A vertical incision was made in the middle of the lower part of the scrotum. After cutting into the superficial perineal muscle layer, the tumor capsule could be seen, which was free along the tumor capsule. The tumor was close to the urethral cavernous body above the tumor, and there were tumor nutrient vessels on the inner side. After carefully ligating the vessels, the tumor was completely removed and observed. The size of the tumor was approximately 8.5 cm × 6 cm × 5 cm in size (Figure 2A); the specimen was soft. The surface was off-white, jelly-like, and rubbery after cutting (Figure 2B). The pathological report from our hospital shows that under the microscope, there were abundant interstitial mucus, fibroblasts, more thin-walled vascular hyperplasia, and no epithelial cells (Figure 3). Based on these pathological findings, the diagnosis of perineal SA was confirmed. The patient recovered successfully and was discharged on the fourth postoperative day, and is still being followed up with no recurrence, and he expressed great satisfaction with the surgical treatment.

## Discussion

SA is a rare benign skin tumor, which is considered a special type of soft tissue tumor. They are difficult to diagnose because they lack unique features like fibroepithelial polyps (3). In recent years, SA has been clearly defined as an isolated soft tissue tumor entity. They can occur anywhere in superficial tissue and are painless (4), slow-growing masses. Allen et al. first reported 28 cases of SA in 1988 and named it superficial vascular mucinous adenoma (5). The clinical manifestations are mainly skin papules, nodular or polypoid masses, no pain, and a wave motion on palpation. The skin color on the surface is normal and mostly single lesion (6). In 2002, the World Health Organization classified SA as a benign tumor with undetermined differentiation (6). SA is rare in clinic and has not been reported in a large sample volume. At present, more than 30 cases have been reported in China. There is no significant gender difference in the incidence population, and the incidence is slightly higher in men than in women (4). The peak incidence is at the age of 40 years approximately (7). The disease can occur throughout the body (4, 8), mainly in the trunk, extremities, head and neck, and rarely in the perineum and vulva (3, 9). This case is a middle-aged man with a giant tumor in the perineum, which is rare.

Similar to other interstitial masses in the perineum, SAs are cystic in nature and are easily misdiagnosed as polyps or epidermoid cysts during physical examination (10). The surface of the SA is covered with epithelial tissue. It has a white, gray appearance, with occasional bleeding due to skin abrasion. It is an enveloped, soft, lobulated cystic tissue. The cut surface of the mass is shiny, colloid-like, translucent, gel-like tissue (2). The same is true for the surface of the mass incision in this case. Its pathogenesis is not yet clear. SAs may be sporadic or related to Carney syndrome, which is an autosomal dominant syndrome characterized by cardiac and mucosal skin myxoma, skin pigmentation, and a variety of endocrine gland hyperthyroidism (mainly endocrine adenoma) (11). This case is an isolated perineal SA in a man. No cardiac myxoma or other endocrine abnormalities were found during the preoperative examination.

The preoperative diagnosis of SA is difficult, and imaging helps to clarify the relationship between the SA and the surrounding tissues. On ultrasound, the SA appears as a confined round or oval mass with heterogeneous internal echogenicity. The CT/magnetic resonance imaging (MRI) scan of SA mostly shows a rounded/lobular soft tissue mass with well-defined borders and superficial lesions, with hypodense/low signal foci without significant enhancement. Its substance is the mucus-rich stromal component of the tumor. The separation of collagen bundles is also seen, showing isointensity (CT)/low signal (MRI). The literature reports a slightly dense or mixed signal with focal hemorrhage (12).

Clinically, SA usually has good boundaries and often extend to subcutaneous fat (12). Histologically, SA has an extensive mucus stroma consisting of loose spindle or stellate fibroblast-like lobular aggregates with an abundant mucus stroma containing thin-walled, medium-sized, hyaline vessels in a disorganized arrangement (13). The nuclei are ovoid, slightly darkly stained or vesicular, with inconspicuous nucleoli, no obvious heterogeneity, and rare nuclear division. In addition, a small number of inflammatory cells, mainly lymphocytes, neutrophils, and eosinophils, can be seen (14, 15). The presence of neutrophils can be a diagnostic clue, especially in the absence of skin ulceration or inflammation, as neutrophils are not present in other mucinous lesions of the skin. SA should be distinguished from all malignant and benign myxoid tumors on the surface, including aggressive angiomyxoma, myxoid neurofibroma, dermal nerve sheath myxoma, and low-grade fibromyxoid sarcoma.

Complete surgical excision is the best treatment for superficial hemangio mucinous tumors. Although SA is a benign tumor of the skin, there is a 30%–40% chance of local recurrence after surgery due to incomplete excision or blurred margins (16). A regular review and close follow-up should be done after surgery. No distant metastasis or malignancy has been reported so far (17).

## Conclusions

The incidence of SA is low and the clinical presentation lacks specificity, but SA should be considered when painless pelvic and perineal swellings without other features are found in young and middle-aged men. A pathological examination is the gold standard for diagnosing this disease. However, the relationship between the mass and the surrounding tissues and whether it invades deep tissues can be clarified by ultrasound, MRI, and CT auxiliary examination, which is a reliable guide for judging the benignity and malignancy of the tumor and its prognosis. Extensive surgical excision is currently the main method of treatment, ensuring that the edges of the mass are removed intact to avoid postoperative recurrence. Although SA has a good prognosis, patients still need to be informed of regular follow-ups.

## Data Availability

The raw data supporting the conclusions of this article will be made available by the authors, without undue reservation.
